# Research Frontiers of Health Emergency and Disaster Risk Management: What Do We Know So Far?

**DOI:** 10.3390/ijerph17051807

**Published:** 2020-03-10

**Authors:** Emily Ying Yang Chan, Holly Ching Yu Lam

**Affiliations:** 1 JC School of Public Health and Primary Care, Faculty of Medicine, The Chinese University of Hong Kong, Hong Kong, China; 2 Nuffield Department of Medicine, University of Oxford, Oxford OX3 7BN, UK; 3 Genomic and Environmental Medicine, National Heart and Lung Institute, Imperial College London, London SW7 2AZ, UK; ching.lam@imperial.ac.uk

Health-Emergency Disaster Risk Management (Health-EDRM) emerged as the latest knowledge, research and policy paradigm shift from response to preparedness and health risk management in non-emergency times [1]. This approach attempts to enlist and empower communities to invest and emphasize their disaster health risk reduction efforts, thereby strengthening health systems and supporting community health resilience building. This Special Issue has collected 20 scientific papers that attempt to examine the research frontier in Health-EDRM.

Major Health-EDRM research evidence gaps were found during a global research agenda setting meeting of the 2018 WHO Health-EDRM global research group in Kobe. Kayano, Chan, Murry et al. [2] highlighted the development need for relevant research methodologies, risk communication approaches, health data management strategies, practical health emergency study ethical guidelines, bridging global research capacity disparities, and infrastructure constraints, to ensure knowledge advancement in Health-EDRM. The authors also pointed out the lack of understanding of psychosocial health risk profiling in population subgroups. Reifel’s analysis showed a better understanding of current doctrines and practices in both clinical mental health practices and policy, which will help to bridge the conceptual interlinkages between the preventive-based disaster risk reduction policy agenda and the curative-focused disaster mental health discipline [3]. Genereux, Schluter, Tamahashi et al. [4] argued that standardizing psychometrically robust instruments would also be urgently needed to identify at-risk patients throughout—before, during, and after emergencies and disasters—to ensure that mental and social health needs are addressed throughout the pathway of care (prevention, screening, diagnosis, treatment, and rehabilitation). Aung, Murry and Kayano [5] discussed the need for new research and ethical guidelines to harmonize research efforts in Health-EDRM and Kubo, Yanasan, Herbosa et al. [6] described the challenges in the standardization of health data collection throughout the research processes.

Existing surveillance databases, new study tools, and innovation methodologies may help to identify population health risks and support Health-EDRM policy development. Using a syndromic surveillance database in the Philippines, Salazar, Law, Winkler [7] showed how an existing clinical based database might be useful in assisting emergency health service planning decision making during outbreaks in armed conflict. Even with the database limitations to report injuries and death, this existing data system was nevertheless useful to support non-communicable diseases’ service caseload planning. Using the computerized random digit dialling methods for rapid data collection after a major urban subway fire incident, Chan, Huang, Hung et al. [8] captured health risk perception, misconceptions, and community first-aid response knowledge in urban man-made emergency incidents. Using social media data from Twitter, Gruebner, Lowe, Sykora et al. [9] showed how the spatio-temporal distribution of negative emotions varied in New York City after a natural disaster. Their study showed that pre-disaster status could be used as a significant predictor of post-disaster emotional outcomes in communities. Using a disturbance management model to estimate logistics constraints in medical supplies during natural disasters, Shi and He [10] examined how medical supplies, which required cold-chain support (e.g., blood and vaccines), might be optimized after natural disasters when transport might be disturbed. Using a three-phase methodology and the online, global, and publicly available databases, Chan, Huang, Lam et al. [11] developed a health vulnerability index (HVI) that captures seven main health dimensions with nine indicators. This index allows the inclusion of non-communicable disease burden of countries/communities into the disaster risk assessment and may reflect underlying health needs and the capacity requirement to address Health-EDRM at the country level.

This Special Issue also presents studies that attempted to capture health consequences of less reported extreme events and identify at-risk communities. With the increased frequency of climate-induced disasters and weather events [12], household preparedness is regarded as an important means of bottom-up resilience. Yet, limited research evidence is currently available to understand how climate events might affect megacities in Asia. Chan, Man, Lam et al.’s [13] paper of health risks and impact after the 2018 Super Typhoon Mangkhut in Hong Kong SAR China showed that education status, risk perception, routine household emergency preparedness, and previous experience of direct disaster impact are factors associated with the uptake of typical typhoon-specific preparedness measures (TSPM). Belleville, Ouellet, Morin [14] documented the post-traumatic stress symptoms reported and experienced by evacuees after the 2016 Fort McMurray wildfires in Canada. Their study findings showed that a significant proportion of the study participants reported post-traumatic stress symptoms that might warrant clinical attention and argued that the mental health at-risk population should be identified to protect psychosocial well-being after large-scale disaster events.

Translation of research evidence to programme and policy agenda has been a major constraint to ensure evidence-based practices. Genereux, Lafontaine and Eukelbosh [15] identified some key determinants that facilitate knowledge-to-action strategies for better community health risk preparedness. The team argued that blending traditional and modern approaches, fostering community engagement, cultivating relationships, investing in preparedness and recovery, putting knowledge into practice, and availability of human and financial resources are key successful factors for integrating expertise and research in disaster management practice. Yet, a lack of emergency preparedness often exacerbates the underlying community health risks in resource-deficit minority-based areas in times of crisis and emergencies. Ho, Chan, Lam et al. [16] showed indicators capturing perceived water security in the non-emergency/normal period might not be associated with Health-EDRM preparedness attitude and coping ability in a water-stressed rural context of PRC China. Chan, Lam, Lo et al. [17] showed food-labelling and perceptions of food-related health risk might be influenced by face-to-face health education interventions and should be considered as a core emergency preparedness and health risk reduction management strategy to strengthen bottom-up resilience in minority communities. Kamara, Akombi, Agho et al. [18] conducted a systematic review of resilience and well-being evidence in southern Africa and showed disaster risk reduction interventions that were based only on Western modelled or scientific warning systems might undervalue traditional warning insights and undermine intrinsic and community capacities. To strengthen resilience and well-being outcomes, efforts should be invested to ensure household, community/indigenous knowledge, and government-level capabilities are harnessed. Public health planning models might be instrumental in facilitating the planning for Health-EDRM-related risk reduction efforts in both emergency and non-emergency situations [19,20].

Perhaps one of the most important global health emergency incidents for 2020 would be the global response to COVID-2019 [21]. With their proposed influenza A simulation model that considered the three main routes of transmission—long–range airborne, fomites, and close contact—Zhang and Li [22] found in a non-clinical office setting that mask wearing and regular cleaning of high-touch surfaces were more useful than hand-washing for viral-related disease transmission control. Through their retrospective public health response policy analysis of SARS and MERS in South Korea, Lee and Jung [23] showed that legislation and leadership influenced the overall emergency response process and the establishment of intergovernmental response systems and the success of risk communications during infectious-related events. Meanwhile, although new technology platforms (e.g., smartphones and internet) had generated interests and showed promises in efficiency and speed in mass communication in emergencies and crises, community receptivity of communication channels might differ with the nature of the emergency events [24]. For major infectious disease-based event such as the H7N9 outbreak in 2014 in Hong Kong, Tam, Huang and Chan [19] showed traditional risk communication channels (television and telephone) might still be the preferred channels of the general public.

New scientific evidence will be generated from the research studies for the 2019 novel coronavirus global epidemic. Not only will such knowledge facilitate a better understanding of new emerging diseases, but also identify treatments, enable a better uptake of community health protection behaviours, examine the usefulness of modern public health measures, and evaluate the effectiveness of technology innovation in health protection; it will also serve to remind researchers, academic, and policy makers that the landscape of Health-EDRM-related research is constantly evolving with global crises and emergencies.
